# A M35 family metalloprotease is required for fungal virulence against insects by inactivating host prophenoloxidases and beyond

**DOI:** 10.1080/21505594.2020.1731126

**Published:** 2020-02-21

**Authors:** Antian Huang, Mengting Lu, Erjun Ling, Ping Li, Chengshu Wang

**Affiliations:** a School of Life Science and Technology, Tongji University, Shanghai, China; b CAS Key Laboratory of Insect Developmental and Evolutionary Biology, CAS Center for Excellence in Molecular Plant Sciences, Institute of Plant Physiology and Ecology, Chinese Academy of Sciences, Shanghai, China; c School of Life Science and Technology, ShanghaiTech University, Shanghai, China; d CAS Center for Excellence in Biotic Interactions, University of Chinese Academy of Sciences, Beijing, China

**Keywords:** *Metarhizium*, metalloprotease, melanization, immune suppression, virulence

## Abstract

A diverse family of metalloproteases (MPs) is distributed in eukaryotes. However, the functions of MPs are still understudied. We report that seven MPs belonging to the M35 family are encoded in the genome of the insect pathogenic fungus *Metarhizium robertsii*. By gene deletions and insect bioassays, we found that one of the M35-family MPs, i.e. MrM35-4, is required for fungal virulence against insect hosts. MrM35-4 is a secretable enzyme and shows a proteolytic activity implicated in facilitating fungal penetration of insect cuticles. After gene rescue and overexpression, insect bioassays indicated that MrM35-4 contributes to inhibiting insect cuticular and hemocyte melanization activities. Enzymatic cleavage assays revealed that the recombinant prophenoloxidases PPO1 and PPO2 of *Drosophila melanogaster* could be clipped by MrM35-4 in a manner differing from a serine protease that can activate PPO activities. In addition, it was found that MrM35-4 is involved in suppressing antifungal gene expression in insects. Consistent with the evident apoptogenic effect of MrM35-4 on host cells, we found that the PPO mutant flies differentially succumbed to the infections of the wild-type and mutant strains of *M. robertsii*. Thus, MrM35-4 plays a multifaceted role beyond targeting PPOs during fungus-insect interactions, which represents a previously unsuspected strategy employed by *Metarhizium* to outmaneuver insect immune defenses.

## Introduction

More than 100 families of metalloproteases (MPs) have been identified from different organisms and curated at the MEROPS database [1]. Many MPs are zinc-chelating and play an essential role in microbial pathogenesis [2,3]. For example, a M10 family AprA is a virulence factor of *Pseudomonas entomophila* during bacterial infection of *Drosophila melanogaster* by cleavage and maturation of a bacterial pore-forming toxin [4,5]. AprA is also required for bacterial infection of the bean bugs (*Riptortus pedestris*) by suppressing host cellular and humoral immunities [6]. Different MPs have also been characterized in pathogenic fungi with virulence contributions against different hosts. For example, the M35 family Avr-Pita of the rice blast fungus *Magnaporthe oryzae* was demonstrated as an effector to interact with the host resistance protein Pi-ta inside the plant cells to trigger host defense response [7,8]. In fish pathogenic bacterium *Aeromonas salmonicida*, a M35 MP AsaP1 is also found indispensable for bacterial virulence against fishes [9]. A M36 enzyme UmFly1 is required for the virulence of the smut fungus *Ustilago maydis* by dual cleavage of the maize and fungal chitinases [10]. The M36 family Mpr1 identified from the mammalian pathogen *Cryptococcus neoformans* was found to play a role in invading the central nervous system by targeting brain endothelium [11]. Considering the number and family diversity of MPs, molecular functions of MPs remain understudied. In particular, M35 family MPs that have two zinc-binding histidines and a catalytic residue glutamate in an HEXXH motif are widely distributed in fungi [1]. Except for *Magnaporthe* Avr-Pita, no other M35 MP has been functionally characterized in pathogenic fungi.

Insect pathogenic fungi such as *Metarhizium robertsii* have been used for biocontrol of insect pests and developed as a genetically tractable system for investigation of fungus-insect interactions [12,13]. Both the pathogens and insect hosts have co-evolved with the diverse and well-tuned strategies to outwit each other during the course of interactions. For entomopathogens, serine proteases, chitinases, lipid metabolic enzymes, and insecticidal small molecules are employed by fungi for cuticular penetration and invasion or evasion of host immunities [12]. At the same time, insects protect themselves with the powerful cellular and humoral immune systems to fight off parasite infections [14–16]. It has been known that the activation of the antifungal Toll pathway and prophenoloxidases (PPOs) in insects requires the sequential function of different endogenous serine proteases (SPs) to cleave and maturate the zymogens such as the cleavage of PPOs to POs for melanization responses [17–19]. Insect melanization responses could also be activated by parasite proteases [20]. For example, the infection with transgenic fungi overexpressing a subtilisin SP could severely induce the melanization activity in insects that led to a strong toxigenic effect on both insects and fungal pathogens [21,22]. It has also been found that a thermolysin M4 family LasB from the entomopathogenic bacterium *Pseudomonas aeruginosa* [23] and PrtS from *Photorhabdus luminescens* [24] could induce insect melanization reactions. However, during the course of *Metarhizium* infection, a reduction trend of PO activity was observed in locust [25]. It remains unknown whether the insect pathogen can secrete an effector-like protease to inactivate PO activity and/or inhibit insect antifungal gene expressions to facilitate fungal invasion of host immunities.

About 100 MPs that belong to 25 families are encoded in each genome of *Metarhizium* species [26]. However, the function of MPs has not been well investigated in these entomopathogens. A zinc carboxypeptidase MeCPA (M14 family) of *M. anisopliae* was implicated in degradation of insect cuticles [27]. A recent study indicated that two M43B-type MPs (MrMep1 and MrMep2) are required for fungal development and virulence against insects in *M. robertsii* with unclear mechanisms [28]. Interestingly, it has been found that MP activities (e.g. Mep1 and a M4-type MP) of *M. robertsii* could be induced by insect antifungal peptides and proteinase inhibitors putatively involved in degradation of these host-derived defense molecules [29,30]. Considering that the *Magnaporthe* effector Avr-Pita belongs to a M35 family protein [8], we performed gene deletions of seven M35-domain-containing MP genes in *M. robertsii* in this study and unveiled that one of them is required for fungal virulence against insects by inactivation of insect PPOs and beyond to subvert insect immune responses.

## Materials and methods

### Microbial cultures and growth media

The wild-type (WT) and mutants of *Metarhizium robertsii* strain ARSEF 2575 were maintained on potato dextrose agar (PDA; BD Difco, USA) at 25°C for 14 days in the dark. For RNA extraction, fungal spores were inoculated into Sabouraud dextrose broth (SDB; BD Difco, USA) and incubated in a rotatory shaker at 25°C and 200 rpm for different times. A minimal medium (MM: NaNO_3_, 6 g l^−1^; KCl, 0.52 g l^−1^; MgSO_4_∙7H_2_O, 0.52 g l^−1^; KH_2_PO_4_, 0.25 g l^−1^) amended with 1% (w v^−1^) casein (Sinopharm, China) was used for proteolytic assay. The *Escherichia coli* strain of Trans10 (TransGen Biotech, China) was used for plasmid constructions and BL21(DE3)pLysS (Promega, USA) used for protein expression. The strain AGL1 of *Agrobacterium tumefaciens* was used for fungal transformation.

### Protein distribution and phylogenetic analysis

The M35-domain-containing proteins identified by InterproScan analysis were retrieved from the genomes of different *Metarhizium* species [26] and representative species of bacteria, human and plant pathogenic fungi cataloged in the MEROPs database [1]. Seven M35-domain-containing proteins were identified from *M. robertsii*, and in total, 136 protein sequences were included for phylogenetic analysis in this study. The M35 domain sequences were also retrieved from each sequence for phylogenetic analysis. Signal peptide of each protein was predicted using the program SignalP ver. 5.0 [31]. Both the full protein and M35-domain sequences were aligned using the program ClustalX [32], and the phylogenetic trees were generated using the software MEGA-X with a neighbor-joining method under the parameter control of a Dayhoff model, pairwise deletion of missing data/gaps and 500 replicates of bootstrap tests [33].

### Plasmid construction and gene deletions

To determine the virulence contribution of seven M35 metalloproteases in *M. robertsii*, the genes were individually deleted by homologous recombination. In brief, the 5ʹ- and 3ʹ-flanking sequences of each gene were amplified using the genomic DNA as a template with the Taq DNA polymerase (Vazyme, China) and different primers (Table S1). The fragments were purified, digested with restriction enzymes and then inserted into the corresponding restriction sites of the binary vector pDHt-Bar (conferring ammonium-glufosinate resistance) to produce the deletion vectors for *Agrobacterium*-mediated fungal transformation [34]. For complementation of *MrM35-4* gene deletion, the full-length *MrM35-4* gene including the promoter and 3ʹ-UTR region was amplified and cloned into the binary vector pDHt-Ben (conferring benomyl resistance) and the obtained plasmid was used to transform the null mutant ∆*MrM35-4* for gene rescue. For overexpression of *MrM35-4*, the gene was made under the control of the constitutive *Tef*-gene promoter [35] and the *P_tef_::MrM35-4* fusion cassette was cloned into the plasmid pDHt-Ben to transform the WT strain of *M. robertsii* to obtain the mutant WT::OE. The drug resistance mutants were verified by PCR with different primers (Table S1).

### Phenotyping and stress response assays

To determine if any phenotypic difference between WT and mutant of *MrM35-4*, conidial suspensions (3 μl each of 1 × 10^5^ conidia ml^−1^) were inoculated onto PDA plates. Conidial yields were determined 14 days post-incubation at 25°C. To determine the difference in stress responses between WT and mutants, the strains were cultured on PDA plates amended with the final concentrations of 0.5 M NaCl, 1 M sorbitol, 5 mM CuSO_4_, 1 mM H_2_O_2_, 0.1 mg ml^−1^ Congo red or 0.1 mg ml^−1^ sodium dodecyl sulfate (SDS) for 2 weeks at 25°C. Colony morphologies were photographed. Both the WT and mutant were also inoculated on MM agar containing casein (1%, w v^−1^) for 3 days to compare the caseinolytic ability between WT and mutant strains. Each strain has three replicates per treatment.

### Insect bioassays

To determine the virulence difference between the WT and mutants of *M. robertsii*, insect bioassays were carried out using the last instar larvae of the wax moth (*Galleria mellonella*) as previously described [36]. Topical infection was performed by immersing insects in conidial suspension (1 × 10^7^ conidia ml^−1^) for 1 min. Injection assays were conducted by injecting 20 μl of conidial suspensions (2.5 × 10^4^ conidia/ml) into the insect hemocoel through the second proleg. In addition, both the wild-type strain W1118 and *PPO1^∆^, PPO2^∆^*, and *PPO1^∆^ PPO2^∆^* mutants of *D. melanogaster* [37] were also used for topical infection by using the 1-week-old female adults. There were three replicates for each treatment (15 insects each for wax moth larvae and 30 adults each for *Drosophila*) and the experiments were repeated at least twice. Mortality was recorded every 12 h, and the median lethal time (LT_50_) values were calculated by Kaplan-Meier analysis. During topical infection of the wax moth larvae, additional insects were treated for cuticular penetration and melanization comparison between WT and mutant infections [34].

### Quantitative RT-PCR analysis of gene expressions

To investigate the gene expression profiles, total RNA was extracted from the conidia harvested from the 2-week-old PDA, mycelia from the 3-day SDB, and appressoria induced on locust hind wings for 36 h using the Trizol reagent (TransGen Biotech, China). In addition, the insects were injected with 20 μl of the WT spore suspension (5 × 10^6^ conidia ml^−1^) for 48 h and then bled for harvesting fungal cells (hyphal bodies) by gradient centrifugation using Centricoll (Sigma-Aldrich) at 4°C [36]. This sample was also used for RNA extraction and gene expression assay. First strand cDNA of each sample was synthesized using 1 μg total RNA with the RT Master Mix kit (TransGen Biotech, China) according to the manufacturer’s instructions. The 10-fold dilution of cDNA was used for real-time RT-PCR analysis with a SYBR Mix (Toyobo, Japan). A β-tubulin gene was used as an internal reference [34]. The wax moth larvae injected with fungal spores (20 μl 5 × 10^6^ conidia ml^−1^ per insect) for 36 h were used for fat body isolation and RNA extraction to detect the induction of the antifungal gallerimycin gene transcription in *Galleria* larvae [38]. Following topical infection of *Drosophila* female adults with fungal spores for 36 h, the whole insects were homogenized for RNA extraction and detection of antifungal drosomycin gene expression. The actin 3 gene of *Galleria* (*Gmact3*, MG846876) and ribosomal protein gene *RPL32* (CG7939) of *Drosophila* were amplified as the internal references, respectively.

### Cellophane membrane and cuticle penetration assays

Both the cellophane membrane and locust wings were used for fungal penetration assays. The sterile cellophane membranes were lined on MM agar plates and 3 μl of conidial suspension (1 × 10^6^ conidia ml^−1^) of each strain was inoculated in the middle of the plates for 3 days. The membranes with cultures were then carefully removed and the plates were incubated for another 4 days. The colony sizes were measured and compared between WT and mutants. For cuticle penetration assays, the intact locust hind wings were surface-sterilized in 3% NaClO and washed using sterile water for five times. The wings were then lined on the MM plates [39]. Conidial suspension (1 × 10^6^ conidia ml^−1^; 3 μl each) of each strain was inoculated on the wings for 3 days at 25°C. The wings were then removed and the plates were incubated for four additional days.

### Western blot analysis

To verify the secretion feature of MrM35-4, the polyclonal antibody against MrM35-4 was produced by immunizing rabbits with the predicted and synthesized antigenic polypeptide (CIQLAKRAEQDAKDGS) at the ABclonal Technology Company (China). The actin 3 (GmAct3) of *G. mellonella* was expressed in *E. coli*, purified and used to raise the monoclonal antibody in mouse. The WT strain was cultivated either in SDB or MM amended with 1% (w v^−1^) cuticle (silkworm pupa homogenate) for 3 days. The culture supernatants were collected by filtration and the proteins were precipitated with the addition of (NH_4_)_2_SO_4_ for overnight at 4°C. The precipitates were concentrated by centrifugation at 10,000 rpm for 20 min at 4°C and then reconstituted in 1× PBS buffer (pH 7.4) with the addition of phenylmethylsulfonyl fluoride (PMSF, 1 mM). To detect if any the presence of MrM35-4 in insect hemolymph after fungal infection, conidial suspension of the WT strain was injected into the body cavity of wax moth larvae (5 × 10^6^ conidia ml^−1^, 20 μl per insect). Insect hemolymph was collected with the addition of PMSF and phenylthiourea (to inhibit PPO activity) 12 h, 24 h, 36 h, and 48 h post-injection. Protein concentration was determined using a bicinchoninic acid (BCA) method according to the manufacturer’s instructions (Beyotime, China). For Western blot analysis, the proteins (30 µg of each sample) were separated through a 10% SDS-polyacrylamide gel electrophoresis (PAGE) and transferred to PVDF membranes (Millipore, USA). The membrane was probed using a primary antibody against the MrM35-4 polypeptide antibody (1:3000), and a secondary HRP-conjugated goat anti-rabbit IgG antibody (1:5000). The GmAct3 antibody mentioned above was used (1:3000) as a reference. Immunoblot analysis was visualized using a Chemiluminescence Imaging System (Tanon, China).

### Protein expression and activity assays

To further determine the activity of MrM35-4, the cDNA of *MrM35-4* gene was amplified using a high-fidelity DNA Polymerase (Vazyme, China) and subsequently cloned into the expression vector pGEX-6P-3 for the transformation of the *E. coli* strain BL21(DE3)pLysS. Considering that both PPO1 and PPO2 of *Drosophila* are required for hemolymph melanization [37], these two PPO genes were also cloned into the plasmid pET28a for expressions in *E. coli*. Bacterial cultures were grown in the LB medium and induced with isopropyl β-D-1-thiogalactopyranoside (IPTG) for 16 h at 16°C. Recombinant MrM35-4, PPO1, and PPO2 proteins were purified from bacterial supersonic lysates using affinity chromatographic resins (Beyotime, China). Eluted proteins were desalted with deionized water and concentrated using a 10-kD filter device (Millipore, USA). Protein concentration was determined using the BCA method.

The purified MrM35-4 was further tested to degrade casein (1%) on MM plate for 4 h. *In vitro* activation/digestion of rPPO1 and rPPO2 was tested using MrM35-4, α-chymotrypsin (Macklin, China) and ethanol in a reaction buffer (50 μl) for 30 min at 25°C. The reactions were stopped by adding EDTA (5 mM at a final concentration), PMSF (1 mM) and dopamine (10 mM; Sigma, USA) for 15 min [40,41]. The reaction tubes were photographed. To detect whether rPPO1 can be cleaved byMrM35-4, purified rPPO1 (50 μg) was separately incubated with MrM35-4 (1 μg) or α-chymotrypsin (1 μg) in a 50 μl Tris-HCl buffer (50 mM, pH 7.4) reaction system containing 0.5 mM ZnCl_2_ and 0.5 mM CuSO_4_ at 25°C for 15 min, 30 min, and 45 min [40]. The samples were then mixed with the loading buffer for SDS-PAGE analysis and the gel was stained with Coomassie blue. The hemolymph of *G. mellonella* was also collected on ice for degradation analysis. After adding phenylthiourea, the sample was centrifuged at 4°C for 5 min. The hemolymph proteins (100 μg each) were then treated with or without rMrM35-4 (1 μg) in a 50 μl Tris-HCl buffer for 1 h and the samples (20 μl each) were analyzed by SDS-PAGE analysis.

### Induction of S2 cell apoptosis assay

To test whether MrM35-4 can mediate an apoptogenic effect, *Drosophila* S2 cells were treated with the protease. The cells were cultivated in the Schneider’s *Drosophila* medium (Gibco, USA) at 27°C in the dark and the purified protein MrM35-4 was added at a final concentration of 10 μg ml^−1^. After incubation at 27°C for 3 h, cells were collected by centrifugation at 1000 g for 5 min and resuspended in 450 μl 1× PBS buffer (pH 7.4). Subsequently, 50 μl of 0.5% trypan blue (Solarbio, China) was added into the cell suspensions for 10 min. The dye can be taken up by damaged and dying cells [42]. The stained cells were directly observed under a microscope and the dying cells were counted. In addition, the induction of S2 cell apoptosis after enzyme treatment was tested using the Apoptosis Detection Kit (Beyotime, China). Briefly, the S2 cells were treated with MrM35-4 (at a final concentration of 10 μg ml^−1^) at 27°C for 1 h. The cells were collected, washed with 1× PBS buffer (pH 7.4), and stained in the binding buffer (100 μl) with the addition of 2 μl Annexin V-FITC stock solution (BD Bioscience, USA) and 5 μl propidium iodide (PI) stock solution [43]. The cells were incubated at room temperature for 30 min in the dark. After staining, the cells were transferred onto the slides and gently rinsed with 1× PBS buffer (pH 7.4). The stained cells were observed under a fluorescence microscope. Each experiment had three independent replicates and the Student’s *t*-test was used to compare the differences between treatments.

## Results

### M35 metalloprotease distribution and phylogenetic analysis

Our genome survey indicated that seven M35-domain-containing MPs with different sizes are encoded in the genome of *M. robertsii*, termed MrM35-1 to MrM35-7. *In silico* analysis suggested that only MrM35-6 has no signal peptide whereas the other six MPs are putatively secretable (Figure S1). Further analysis indicated that two to six of M35-family MPs are encoded in different insect pathogenic fungi. Varied numbers of MPs are also present in plant and mammalian pathogenic fungi as well as a few bacterial species. In contrast, M35-type MP is not present in plants and animals including different insects (Figure S2). The protein sequences of 136 M35-family MPs were retrieved from selected fungal and a few bacterial species and used for a phylogenetic analysis. As a result, at least 10 subfamilies (SFs, each at least containing more than two proteins) could be obtained in a neighbor-joining tree (Figure S3). The SF1 lineage only contains MPs from the dermatophytes whereas SF6 only for plant pathogens and SF10 specific for insect pathogens. For seven M35 MPs encoded in *M. robertsii*, MrM35-2 and MrM35-4 are closely related to each other and clustered together (SF8) with the *Magnaporthe* Avr-Pita effector [8]. The M35 domain sequences were also retrieved from each sequence for phylogenetic analysis, and at least nine domain-specific subfamilies (dSFs) were obtained (Figure S4). The topologies of these full-sequence and domain-specific trees were largely incongruent to each other. For example, in contrast to the full-sequence tree, the M35 domain of MrM35-4 and its counterparts formed a basal lineage (dSF9) while the dSF2, dSF3, and dSF4 clusters did not contain the domain sequence from *Metarhizium*. The heterogeneity among these M35 MPs would suggest functional divergence of these enzymes between and within fungal species.

### Gene deletions and identification of a virulence-related MP gene

**Figure 1.**
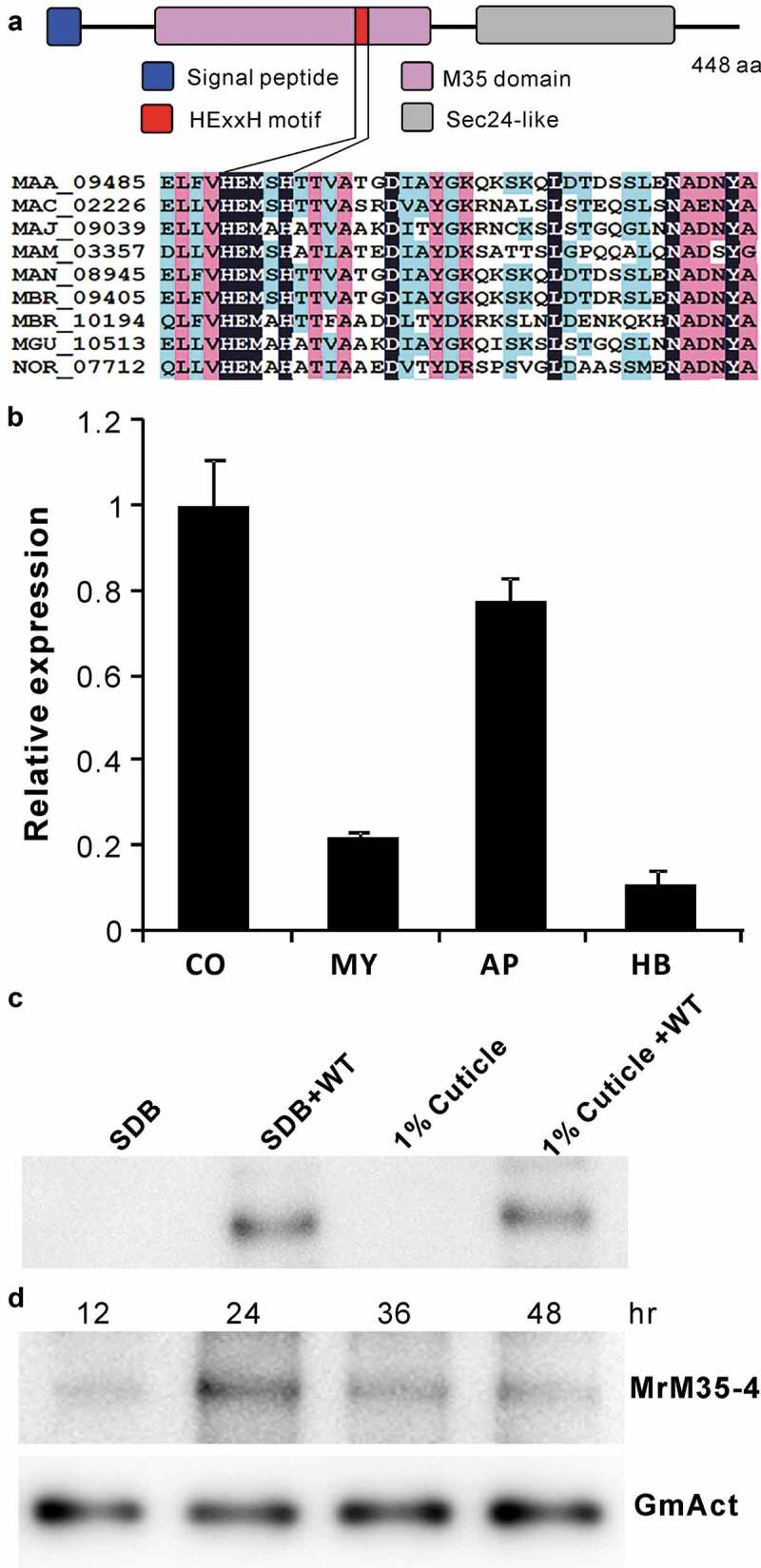
Schematic structure and expression analysis of the metalloprotease MrM35-4. (a) Schematic structuring of the enzyme. The conserved domain and motif are indicated. (b) Transcription of *MrM35-4* by the fungus at different stages. RNA was extracted from different samples including: CO, conidia harvested from 2-week-old PDA plate; MY, mycelia harvested from 3-day-old SDB culture; AP, appressoria induced on locust wings for 36 h; HB, hyphal-body cells harvested from the hemocoel of wax moth larvae 48 h post-injection with fungal spores. (c) Protein expression and secretion analysis. The WT spores were inoculated in SDB or MM medium containing 1% silkworm pupa homogenate for 3 days, and the proteins concentrated from the supernatants were used for western blot (WB) analysis with the polyclonal antibody raised against MrM35-4. The uninoculated SDB and 1% cuticle samples were included as mock controls. (d) *In vivo* detection of the enzyme in insects after injection with fungal spores for different times. The monoclonal antibody raised against the GmAct3 of *G. mellonella* was used in WB analysis as a reference.

To determine the virulence contribution of these MPs in *M. robertsii*, seven genes were individually deleted and verified (Figure S5). The obtained mutants were used for bioassays against the last instar larvae of wax moth (*Galleria mellonella*). The results indicated that, relative to the wild type (WT), only the deletion of *MrM35-4* led to a significant (*P* = 0.001) reduction of fungal virulence (Table 1). Thus, MrM35-4 was further investigated to determine its mechanistic contribution to fungal virulence. This protein contains a signal peptide and the M35 domain harboring the conserved HEXXH motif for zinc chelating, the typical feature of the zincin-type MPs [1,3]. Its C-terminus contains a putative Sec24-related domain (Figure 1(a)). Quantitative gene expression analysis revealed that *MrM35-4* could be more highly transcribed by the fungus in conidial and appressorial cells when compared with the cells grown in an artificial medium or propagated in insect hemolymph (Figure 1(b)). After growing the WT in liquid media, the protein could be detected in the concentrated supernatants by western blot analysis (Figure 1(c)), confirming that MrM35-4 is a secretable protein. The protein could also be detected in insect hemolymph after injection of insects with fungal spores for different times (Figure 1(d)).

**Table 1.** Insect bioassays against the wax moth larvae.

Isolates	LT_50_ (days)	χ2	P value*
WT	3.822 ± 0.133	–	–
Δ*MrM35-1*	3.856 ± 0.128	0.063	0.801
Δ*MrM35-2*	4.044 ± 0.178	0.833	0.361
Δ*MrM35-3*	4.122 ± 0.199	1.280	0.258
Δ*MrM35-4*	4.589 ± 0.137	10.573	0.001
Δ*MrM35-5*	4.133 ± 0.189	1.810	0.178
Δ*MrM35-6*	3.889 ± 0.135	0.099	0.753
Δ*MrM35-7*	3.667 ± 0.090	0.735	0.391

*, comparison was made between WT and individual mutant.

**Figure 2.**
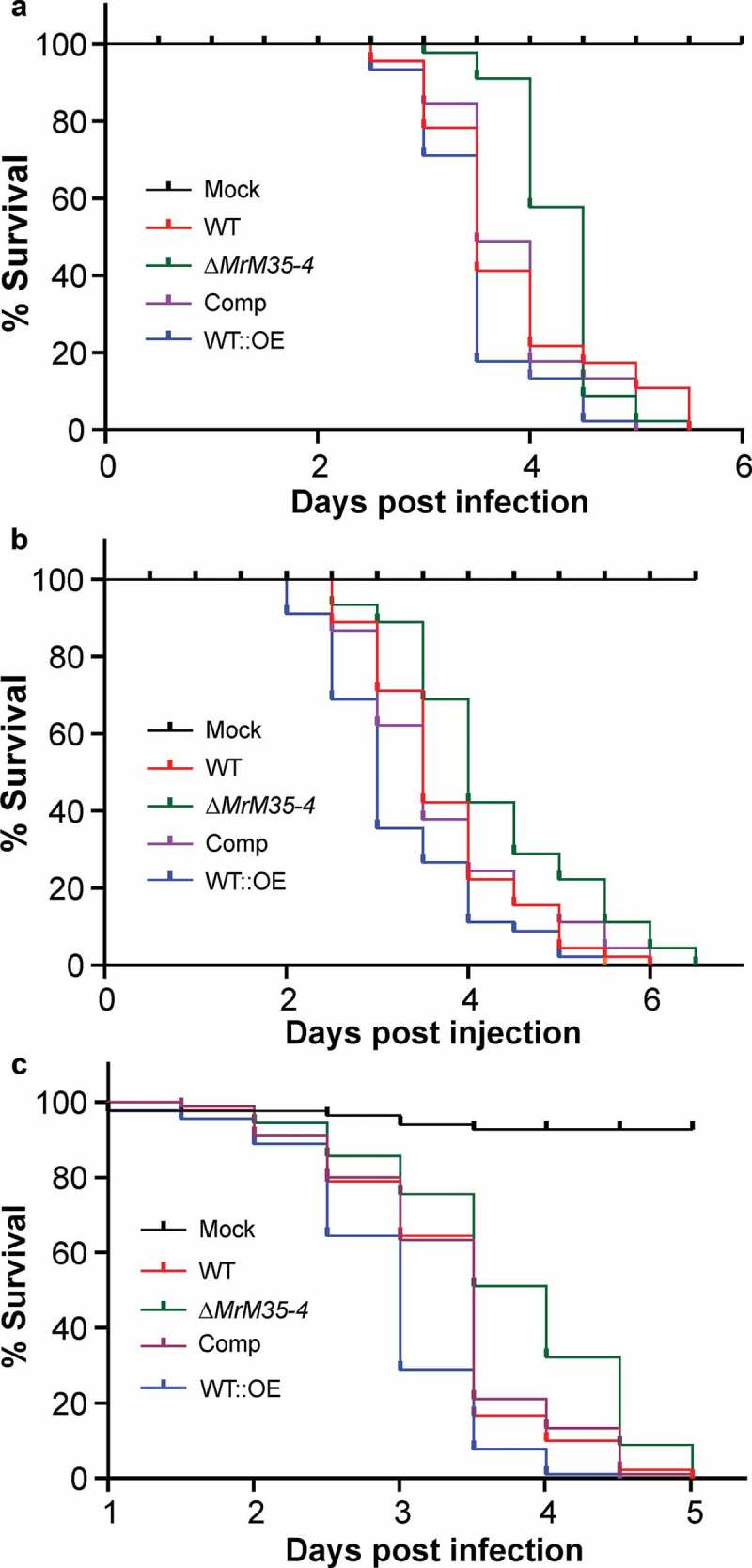
Insect survivals. (a) Survival of the wax moth larvae after topical infection with the WT and mutant strains. (b) Survival of the wax moth larvae after injection with the spores of the WT and mutant strains. The last instar larvae of wax moth were used for bioassays. (c) Survival of the wild-type *D. melanogaster* after topical infection with the WT and different mutants of *M. robertsii.*

To further verify the virulence contribution of MrM35-4, null mutant was complemented with the cassette containing the authentic promoter and coding sequence of *MrM35-4*, and the gene was also overexpressed in the WT strain to obtain the mutant WT::OE under the control of a constitutive promoter (Figure S5). In contrast to the null mutants of M43B genes of *M. robertsii* [28], deletion of *MrM35-4* had no obvious negative effect on fungal growth on PDA plates (Figure S6) or fungal stress responses against the osmotic and oxidative conditions or cell wall biosynthesis inhibitors (Figure S7). Additional insect bioassays were performed by including the gene-rescued and overexpression mutants against the wax moth larvae. The results confirmed that deletion of *MrM35-4* could significantly reduce fungal virulence during both topical infection (χ^2^ = 6.28, *P* = 0.0122) and injection (χ^2^ = 8.27, *P* = 0.004) assays when compared with the WT strain. In contrast, overexpression of *MrM35-4* could substantially increase fungal virulence during both topical infection (χ^2^ = 5.699, *P* = 0.017) and injection (χ^2^ = 6.854, *P* = 0.0088) assays (Figure 2(a,b)). Topical infection of the female adults of *D. melanogaster* resulted in a similar result that deletion of *MrM35-4* could significantly reduce fungal virulence (χ^2^ = 15.864, *P* = 6.8e-5) while gene overexpression increased fungal killing speed (χ^2^ = 18.205, *P* = 2e-5) when compared with the WT (Figure 2(c)). MrM35-4 is therefore a virulence factor of *M. robertsii*.

### MrM35-4 is required for insect cuticle penetration

**Figure 3.**
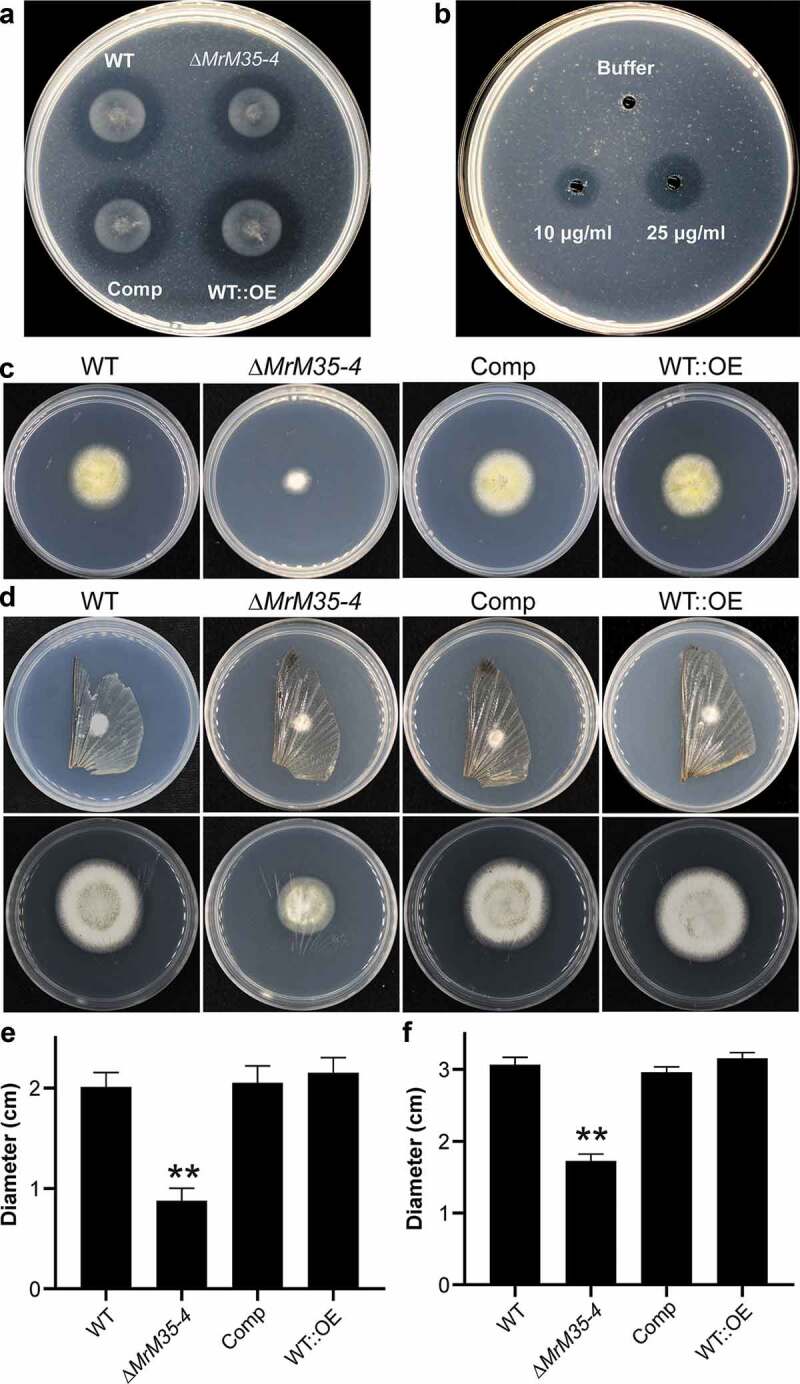
Comparative proteolytic and penetration assays between WT and mutant strains. (a) Casein degradation. Spore suspension (3 μl of 1 × 10^6^ conidia ml^−1^ each spot) was inoculated on MM medium containing 1% casein for 3 days. (b) Caseinolytic assay with the purified protease. Different amounts of MrM35-4 were loaded in MM medium containing 1% casein for 4 h. (c) Cellophane membrane penetration assays. The phenotypes of the WT and mutants grown on the MM medium after the inoculated cellophane membranes were removed for 4 days. (d) Locust wing penetration assays. The locust hind wings lined on the MM medium were inoculated in the middle for 3 days (top panels) and then removed. The plates were kept for incubation for another 4 days (lower panels). (e, f) Comparative analysis of the colony diameter size after removing the cellophane membranes (e) or locust hind wings (f) for 4 days. The difference was compared between WT and individual mutant. **, *P* < 0.01.

We examined the proteolytic effect of the WT and mutants on a casein-containing medium. Based on the formation of hydrolytic zone size 3 days post-inoculation, we found that deletion of *MrM35-4* could considerably (*t*-test; *P* = 0.036) reduce fungal ability to digest casein when compared with the WT. In addition, relative to that of the WT, the hydrolytic zone size formed by WT::OE was significantly (*P* = 0.025) increased (Figure 3(a)). MrM35-4 was also successfully expressed in *E. coli* (Figure S8A), and the enzyme ability to digest casein was further verified in an amount-dependent manner (Figure 3(b)).

Fungal penetration of protein- and chitin-rich insect cuticles is mediated jointly by enzymatic hydrolyses and cellular turgor pressure generated in infection cells [12,44]. We further examined whether the deletion or overexpression of *MrM35-4* would affect fungal penetration ability. The fungi were first determined on penetration of cellophane membranes. As a result, we found that deletion but not overexpression of *MrM35-4* impaired fungal penetration ability (*P* = 0.009) when compared with the WT (Figure 3(c,e)). The fungi were also inoculated on the locust hind wings. Relative to the WT, ∆*MrM35-4* was also found with a reduced ability (*P* = 0.0033) for penetration. However, there was no apparent difference between WT and WT::OE mutant (Figure 3(d,f)). Thus, the effect of MrM35-4 on cuticular penetration would contribute, at least in part, to fungal virulence.

### Suppression of insect immune responses

**Figure 4.**
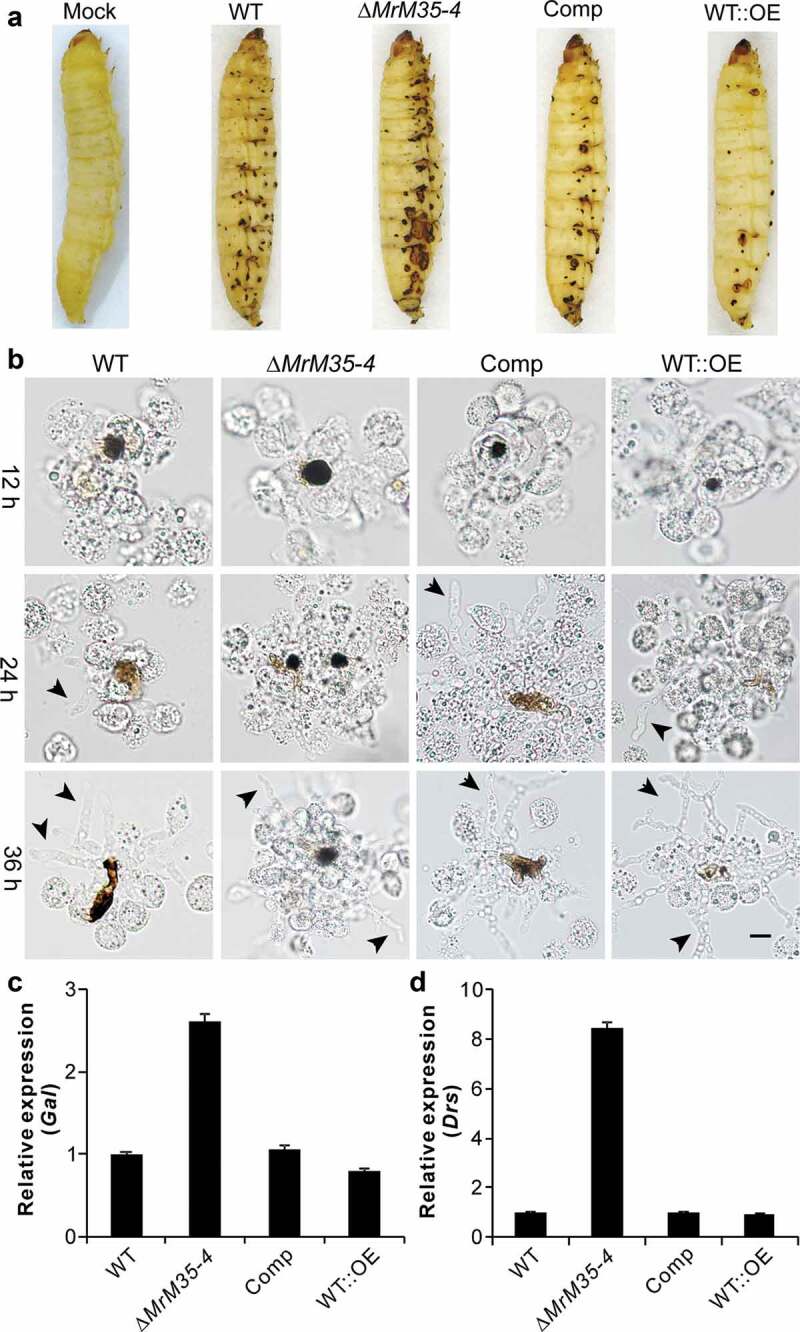
Suppression of insect immune responses. (a) Cuticular melanization of wax moth larvae. The insects were immersed in the spore suspension of each strain for 30 sec and photographed 48 h post-inoculation. (b) Hemocyte encapsulation and melanization. The wax moth larvae were injected with the spore suspensions of each strains and individual insects were bled at different time post-injection to observe insect immune responses against different strains. Fungal cells escaped from hemocyte attack are arrowed. (c) Expression of the antifungal galllerimycin (*Gal*) gene in wax moth larvae after infection with WT and mutant strains. The wax moth larvae injected with fungal spores for 36 h and the fat bodies were isolated for RNA extraction to determine gene expression. (d) Expression of the antifungal drosomycin (*Drs*) gene in *Drosophila* after infection with the WT and mutant strains of *M. robertsii*. The female adults of *Drosophila* were infected and used for RNA extraction 36 h post-topical infection.

We found before that fungal infection could trigger insect cuticular immune responses as manifested by the formation of melanization spots [34,45]. Indeed, the formation of the black spots was observed during topical bioassays using the *Galleria* larvae (Figure 4(a)). Unexpectedly, we found that the infection with ∆*MrM35-4* resulted in a more intensive response of cuticular melanization whereas the insects infected with WT::OE had fewer black spots when compared with those insects treated with the WT spores for 48 h. During injection assays, it was also found that melanization of fungal cells followed by hemocyte encapsulation was increased against ∆*MrM35-4* but substantially weakened against the WT::OE cells when compared with the WT at different time points (Figure 4(b)). We also examined the antifungal gene expressions after topical infection of wax moth larvae and the female adults of *Drosophila* with WT and mutant strains for 36 h. As a result, it was found that ∆*MrM35-4* infection could trigger the upregulation of the antifungal gallerimycin (*Gal*) gene >2-fold in *Galleria* and >8-fold of drosomycin (*Drs*) in fruit flies when compared with the expressions in insects infected by the WT of *M. robertsii*. However, the expressions of both *Gal* and *Drs* had no obvious differences in insects between the WT and WT::OE infections (Figure 4(c,d)). Thus, MrM35-4 is required in *M. robertsii* to inhibit melanization activity and suppress antifungal gene expression in insects. It is noteworthy here that the formation of cuticular melanization spots was unclearly evident during the topical infection of *Drosophila* adults.

### Inactivation cleavage of PPOs by MrM35-4

**Figure 5.**
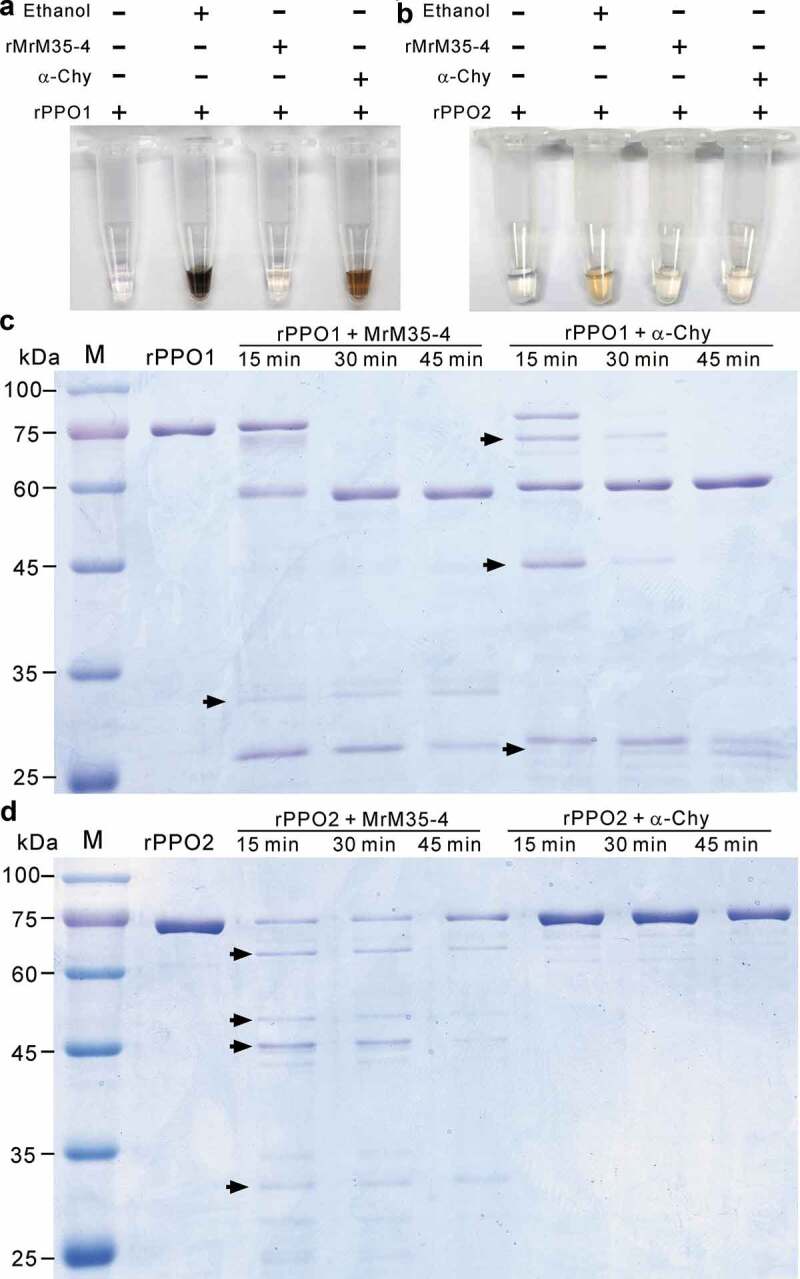
Activity inhibition and degradation of PPOs by MrM35-4. Deactivation of rPPO1 (a) and rPPO2 (b) activity. The reaction buffer (50 μl) contains 50 μg of rPPO1 and 1 μg of either MrM35-4 or α-chymotrypsin (α-Chy) and the tubes were photographed after reaction for 15 min. Comparative degradation of rPPO1 (c) and rPPO2 (d) by MrM35-4 and α-Chy for different times. Arrowed bands show the differences between two enzyme treatments.

The PPO1 and PPO2 produced by crystal cells are responsible for hemolymph melanization after maturation by serine protease cascades in *D. melanogaster* [17,37,46]. To determine the mechanism of MrM35-4 in blocking melanization activity, both PPO1 and PPO2 of *Drosophila* were heterologously expressed and purified (Figure S8B and S8C). We then performed the activation/inactivation experiment of PPO activity using the recombinant MrM35-4 together with ethanol and α-chymotrypsin as positive controls [40]. Both ethanol and α-chymotrypsin could activate rPPO1 activity with the addition of substrate dopamine for 15 min. However, the treatment with MrM35-4 failed to induce melanization reaction (Figure 5(a)). In agreement with our previous finding [41], the melanization activity of rPPO2 was weak when treated with ethanol and α-chymotrypsin, and its activation was also not evident during the co-incubation with MrM35-4 (Figure 5(b)).

To further determine the cleavage difference of rPPO1 and rPPO2 between MrM35-4 and α-chymotrypsin, reaction samples were further analyzed through a polyacrylamide gel electrophoresis. As shown in Figure 5(c,d), we found that the cleavage patterns of both rPPO1 and rPPO2 were substantially different between the treatments with MrM35-4 and α-chymotrypsin after different reaction times. For rPPO1, even the ca. 60 kDa band could be similarly obtained by MrM35-4 and α-chymotrypsin cleavages, there were otherwise variously-sized bands cleaved by each enzyme. The protein rPPO2 was digested into a ladder pattern by MrM35-4 but almost unmodified by α-chymotrypsin. The results revealed therefore that MrM35-4 could clip PPOs in a manner different from the activator of α-chymotrypsin.

### Induction of cell apoptosis by MrM35-4

**Figure 6.**
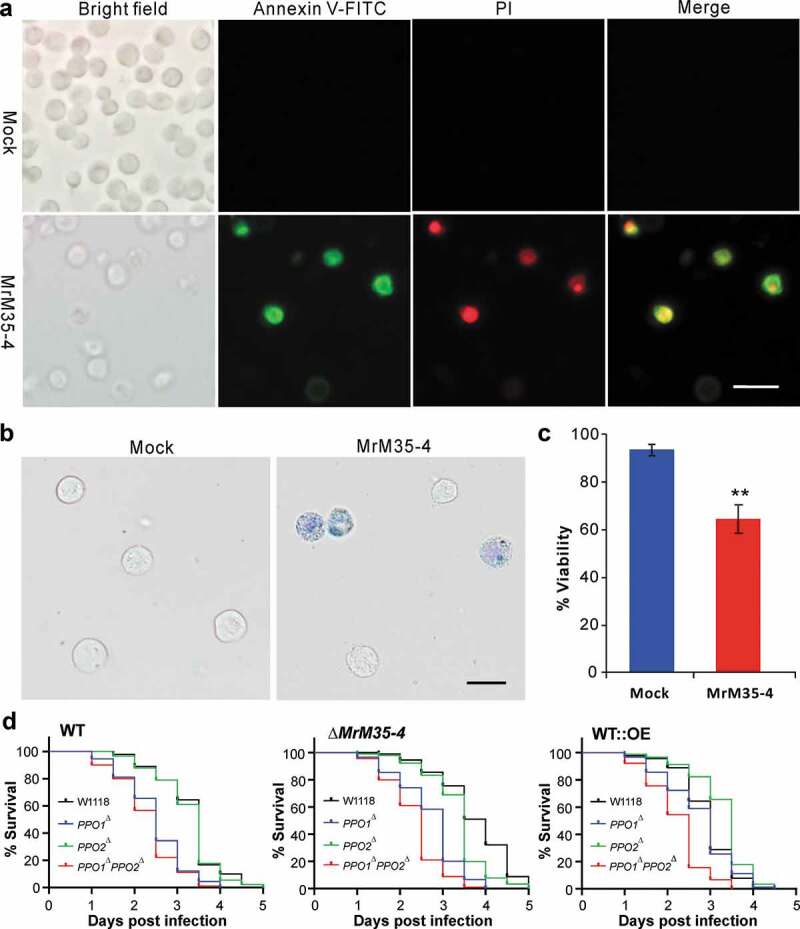
Induction of cell apoptosis and bioassays against different fly strains. (a) Annexin V-FITC staining assay. The S2 cells were incubated with or without MrM35-4 for 1 h and then harvested in PBS buffer by staining with the FITC-conjugated annexin V and PI (propidium iodide) for 30 min in dark. The dying cells could bind annexin V-FITC showing green staining, and those cells that lost membrane integrity could be stained by PI in red. (b) Trypan blue staining. The S2 cells were incubated with or without MrM35-4 for 3 h and then stained with trypan blue for 10 min. (c) Comparison of apoptotic cells between mock control and MrM35-4 treatments. Apoptotic cells were counted after trypan blue staining. **, *t-*test, *P* = 0.0011. (d) Survival of the wild-type, *PPO1*^∆^, *PPO2*^∆^, and *PPO1*^∆^*PPO2*^∆^ mutants of *D. melanogaster* after topical infection with the WT and different mutants of *M. robertsii.*

Bacterial MPs can trigger host cell apoptosis [6,47,48]. To examine whether MrM35-4 has a similar effect, the *Drosophila* S2 cells were treated with the purified protein. Both the annexin V and trypan blue stainings revealed that the co-incubation with MrM35-4 could induce S2 cell apoptosis (Figure 6(a,b)). The viability loss of MrM35-4 treatment was significantly higher (*P* = 0.0011) than that of the mock control (Figure 6(c)). We also found that the cell-free hemolymph protein could be substantially degraded by rMrM35-4 (Figure S8D). Based on these understandings, the fruit-fly mutants of *PPO1^∆^, PPO2^∆^*, and *PPO1^∆^PPO2^∆^* together with the wild-type strain W1118 were used for further bioassays with the WT and mutants of *M. robertsii*. The results indicated that the survival of the mutant and wild-type flies varied substantially when infected by either the WT, ∆*MrM35-4* or WT::OE mutant of *M. robertsii* (Figure 6(d); Table S2). In particular, the double mutant *PPO1^∆^PPO2^∆^* could be more (*P* < 1.0e-10) quickly killed by either the WT, ∆*MrM35-4* or WT::OE when compared with the W1118 flies. Otherwise, *PPO1^∆^* flies were susceptible to the WT and ∆*MrM35-4* but not to WT::OE of *M. robertsii* whereas *PPO2^∆^* flies behaved more or less similar to W1118 when infected by the WT and mutant strains. Thus, in addition to confirming that PPOs are required for fly survival to fungal infection [37], the data revealed that MrM35-4 is required for fungal virulence beyond the targeting and inactivation of PPOs.

## Discussion

Metalloproteases characterized in pathogenic fungi were largely found with the abilities to degrade host tissues [49]. In this study, we found that one of seven M35 MPs encoded in the entomopathogenic fungus *M. robertsii* is required for full fungal virulence against insect hosts by facilitating cuticle penetration, inactivating host PPOs, induction of cell apoptosis and suppression of antifungal gene expression. Considering that the homolog of MrM35-4 is widely present in different *Metarhizium* species and other insect pathogenic fungi, the obtained data would suggest a previously unsuspected strategy employed in fungal entomopathogens to invade insect immune responses. Since the full sequence and M35 domain-specific trees are not congruent with each other, functional evolution of these MPs might not be solely associated with the conserved M35 domains. The exact function of other M35 MPs remains to be determined in *M. robertsii*.

A previous report indicated that the isoforms of MPs could be released during hydration of *Metarhizium* conidia [50]. Somehow consistent with this notion, we found that *MrM35-4* was highly expressed in conidial spores and during fungal formation of the infection structure appressoria. Taken together with the secreted feature of MrM35-4, this MP might be a spore-bound protease that could facilitate fungal infection at an early stage. Consistently, different MPs have also been detected in the cell walls of different cell types of the entomopathogen *Beauveria bassiana* [51]. It has been found that the nutrient contents of the *Metarhizium* growth media affected the conidial yields as well as the activity of spore-bound serine proteases [52]. It remains to be determined whether MrM35-4 expression and accumulation are also associated with fungal growth conditions.

We found that MrM35-4 could be detected in the hemolymph of *Galleria* after injection of *Metarhizium* spores. Interestingly, it has been found before that insect MP inhibitors (IMPIs) could be induced in *Galleria* upon the infection of *B. bassiana* [53] or *M. robertsii* [29]. It has also been demonstrated that injection of a sublethal dosage of microbial thermolysin (M4) could induce the production of an IMPI in the hemolymph of *Galleria* larvae [30]. Both in *Drosophila* and human, a family of MP inhibitor has been identified as the regulator of matrix MPs that play an essential role in determining extracellular matrix structure, cell adhesion, and growth factors [54]. Since the inactivation of *Galleria* melanization activity by MrM35-4 could occur at both cuticle penetration and hemocyte encapsulation stages, the IMPI(s), if any, produced by insects might not be able to inhibit the activity of MrM35-4. Alternatively, except for the inhibitor specificity issue, it cannot be ruled out that the insect pathogens such as *M. robertsii* evolved with an additional mechanism to suppress or inactivate the IMPIs produced by insects. However, this requires further investigation in the future.

Different mechanisms have been found in entomopathogenic fungi to evade or invade insect immune responses. For example, a collagen-like coat protein is deployed on the cell surface of *Metarhizium* to camouflage the cell wall components from insect immune recognition [55]. Chitin-binding LysM effectors can be transcribed by *B. bassiana* to protect fungal cell walls and to suppress insect immunities [36]. A sterol carrier protein horizontally acquired from insect hosts in *Metarhizium* can facilitate the dispersal of fungal cells in the host body cavity [56,57]. It has also been found that small molecules such as destruxins, cyclosporins, and oosporein as well as the small RNAs produced by entomopathogens can inhibit insect cellular and/or humoral immune responses upon fungal infections [38,58–60]. In particular, oosporein and destruxins have been demonstrated with the abilities to inhibit insect PPO activation [38,59]. In addition to facilitating cuticle penetration, we found in this study that MrM35-4 of *M. robertsii* could inactivate PPOs by alterative cleavage that resulted in the patterns differing from the activation clipping of PPOs by serine proteases. We demonstrated before that the N- and C-termini of rPPO1 were sequentially cleaved by α-chymotrypsin to produce an active rPO1 (ca. 60 kDa). In particular, two amino acid bonds ^52^RF^53^ and ^164^RD^165^ of PPO1 were found to be the key cleavage sites for obtaining the active rPO1 [40]. The cleavage sites of PPO1 and PPO2 by MrM35-4 remain to be determined in the future.

Relative to the avirulent bacterial strain, suppression of antimicrobial gene expressions is the characteristic feature of virulent strain against *Drosophila* [61]. Biochemical studies in different insects such as *D. melanogaster, Manduca sexta* and *Tenebrio molitor* have also shown that melanization is connected with the activation of the antifungal Toll pathway by sharing common upstream regulatory serine proteases [17,46,62]. In addition to inactivating PPOs, we found that the expression of the antifungal genes was significantly upregulated in both *Galleria* and *Drosophila* when the insects were infected by ∆*MrM35-4* in comparison to the WT and WT::OE infections, i.e. the involvement of MrM35-4 to downregulate insect antifungal gene expression. It has been shown that a M12B-type VRF1 from the venom of a parasitoid wasp (*Microplitis mediator*) could translocate into the nuclei of the host cells of the cotton bollworm (*Helicoverpa armigera*) to cleave the NF-κB factor Doral to suppress the Toll pathway [63]. We noticed that the M12B family of MPs is also present in the genomes of *Metarhizium* species and other insect pathogens [26,64]. A M85-family AIP56 of the fish pathogen *Photobacterium damselae* could also clip and inactivate the NF-κB factor p65 in host cells to trigger host cell apoptosis [47]. It remains to be determined whether MrM35-4 can enter host cells or not, and how the enzyme directly or indirectly (e.g. through an apoptogenic effect) contributes to suppressing antifungal gene transcription in insects. It has also been found that a M4 MP of *M. robertsii* could be induced by insect proteinase inhibitors and antifungal peptides [29,30]. Functional divergence and substrate specificity between these MPs can be expected, however, remain to be determined. To corroborate the finding that *Metarhizium* infection could induce *Galleria* hemocyte apoptosis or programmed cell death [65], we confirmed the apoptogenic effect of MrM35-4 and that this MP could also degrade insect hemolymph proteins. Future efforts are still required to determine the multifaceted function of these MPs in mediating fungus-insect interactions.

In conclusion, we report the identification and functional characterization of a virulence-related M35-family metalloprotease in suppressing host defense responses in the insect pathogenic fungus *M. robertsii*. The results obtained in this study not only reveal a novel mechanism of fungal pathogenesis but will also promote future investigations of additional MPs involved in fungus-insect interactions.
