# Supplementary material for: A M35 family metalloprotease is required for fungal virulence against insects by inactivating host prophenoloxidases and beyond
Source: Virulence. 2020 Feb 21;11(1):222–37. doi: 10.1080/21505594.2020.1731126 (PMC7051145; doi:10.1080/21505594.2020.1731126)
Supplement: Supplemental Material [file kvir-11-01-1731126-s001.docx]

**Supplementary information**

**Table S1**. Primers used in this study.

| **Primers** | **Primer sequences (5'-3')** | **Restriction enzymes** | **Note** |
| --- | --- | --- | --- |
| MAA_00445UF | GGAATTCCCGGGTCATTTGAAGTCGAG | EcoR I | Gene deletion |
| MAA_00445UR | CGGGATCCATGATGATGGGCTGGGGCTC | BamH I |  |
| MAA_00445DF | GCTCTAGATGCACCGGCAACCAGCTCTC | Xba I |  |
| MAA_00445DR | CGAGCTCTTTCCACAGCTTTGGTACGT | Sac I |  |
| MAA_00445VF | TCAGATGAAACGAAGCGAAGA |  | PCR verification of gene deletion |
| MAA_00445VR | CAGCGGTGATGAGGCTAAAGA |  |  |
| MAA_00460UF | GGAATTCTTGGACGCTGGCATAGCGCT | EcoR I | Gene deletion |
| MAA_00460UR | CGGGATCCTCGCGACAGGGATTCCGAGC | BamH I |  |
| MAA_00460DF | GCTCTAGATCAAGCTACGGCTTGCGCGC | Xba I |  |
| MAA_00460DR | CGAGCTCGCGTTGGCTTACCGGTTAAAAGT | Sac I |  |
| MAA_00460VF | CAGCGGCTAGTTATTTATTTCG |  | PCR verification of gene deletion |
| MAA_00460VR | CCTCTTGACAAGTGACGGAGA |  |  |
| MAA_03242UF | GGAATTCTCAACCCCATACTTGCCATG | EcoR I | Gene deletion |
| MAA_03242UR | CGGGATCCTCTTCTTGCTTTATGCGCATTA | BamH I |  |
| MAA_03242DF | GCTCTAGAGTGGCGGCCCAGGAAGCGGT | Xba I |  |
| MAA_03242DR | GCTCTAGAGTCTTTGATGGCTGGCCATG | Xba I |  |
| MAA_03242VF | AAAAGCCCAATCTTCTCCCT |  | PCR verification of gene deletion |
| MAA_03242VR | CACTGACTCGCAATCTGTTCG |  |  |
| MAA_09485UF | GGAATTCATGATCAACCTAGTCGCGAT | EcoR I | Gene deletion |
| MAA_09485UR | CGGGATCCAGTTCGTGCATTGACAGCAT | BamH I |  |
| MAA_09485DF | GCTCTAGACGAATGACTTTCCCGGTTTTG | Xba I |  |
| MAA_09485DR | GCTCTAGAGTGGAGGCCTCGGGCCTATG | Xba I |  |
| MAA_09485VF | CCCTTGCAGCATAGCCATCT |  | PCR verification of gene deletion |
| MAA_09485VR | GCCTCTGTTCAGGCACTTCG |  |  |
| MAA_09705UF | GGAATTCATGAAGCGAGTTTGGCAGAT | EcoR I | Gene deletion |
| MAA_09705UR | CGGGATCCCTCGGAGCAAACGAATGTTG | BamH I |  |
| MAA_09705DF | GCTCTAGAAGGATATCGTAAAGGACATG | Xba I |  |
| MAA_09705DR | GCTCTAGACAGCGGTCACAGCCTCAGCA | Xba I |  |
| MAA_09705VF | CACCGTCGCCTACTTCACCAG |  | PCR verification of gene deletion |
| MAA_09705VR | GGAACCACCTCGGATAACAGC |  |  |
| MAA_11285UF | GGAATTCGAGGCGCAGGCTTGTCAACT | EcoR I | Gene deletion |
| MAA_11285UR | CGGGATCCGGAGCTGCAGCGGCAACAG | BamH I |  |
| MAA_11285DF | GCTCTAGATGCCAGGGCCAGTATGTAAGC | Xba I |  |
| MAA_11285DR | GCTCTAGAGGACAACACAACAGACGAGGTCG | Xba I |  |
| MAA_11285VF | ACGTATCCCGACCAGAACCT |  | PCR verification of gene deletion |
| MAA_11285VR | CCTTGACACCAACACCCACA |  |  |
| MAA_11678UF | GGAATTCCAACATGAACCAGCCCCTCG | EcoR I | Gene deletion |
| MAA_11678UR | CGGGATCCCGGTAGAGCCACGATTTGTCT | BamH I |  |
| MAA_11678DF | GCTCTAGACTCAGGTCGCTTCTCATCCCG | Xba I |  |
| MAA_11678DR | CGAGCTCCGCCAGACCAACAAGACTTAC | Sac I |  |
| MAA_11678VF | TGTGGAGACTGCCCGAAAG |  | PCR verification of gene deletion |
| MAA_11678VR | CGCCTGATGAGGAGGAGCT |  |  |
| BarF | CGAGTCGACCGTGTACGTCT |  | Validation of marker gene *Bar* |
| BarR | GGTATGACCGGGTCGTTCAC |  |  |
| BenF | CACTCAACATTCAGGCTCCTCT |  | Validation of marker gene *Ben* |
| BenR | GCACAGTAGGCATCAGAGGGAG |  |  |
| MAA_09485CF | AACTGCAGCAAACGACTTGGCTCCTCAG | Pst I | Gene complementation |
| MAA_09485CR | AACTGCAGCCTATGCTCGCTTCCTCACT | Pst I |  |
| MAA_09485CV | AGTGAGGAAGCGAGCATAGG |  | Validation of complementation |
| TefF | TAGCAACAGGCCAGGCTAGACG |  | Amplification of the constitutive promoter |
| TefR | GTTGACGGTTGTGTATGGAAGAT |  |  |
| MAA_09485OEa | GACTAGTTAGCAACAGGCCAGGCTAGACG | Bcu I | Construction of overexpression mutant |
| MAA_09485OEb | GATTCAAAGCCGTAACAAGTCCAGTAAGTCTCATGTTGACGGTTGTGTATGGAAGATTG |  |  |
| MAA_09485OEc | CAATCTTCCATACACAACCGTCAACATGAGACTTACTGGACTTGTTACGGCTTTGAATC |  |  |
| MAA_09485OEd | CGAGCTCCTATTTTCCAGCTGGGACCACCT | Sac I |  |
| rMAA_09485F | GGAATTCGCTGTGCCAATCAACAGCAAC | EcoR I | Prokaryotic expression |
| rMAA_09485R | CCGCTCGAGCTATTTTCCAGCTGGGACCACCT | Xho I |  |
| MrTubQF | GGTCGCTATGAAGGAGGTTGA |  | Real-time PCR |
| MrTubQR | TCCTGGATGGAGGTGGAGTTA |  |  |
| MAA_09485QF | GCAACTTGACACGGACAGCA |  | Real-time PCR |
| MAA_09485QR | CCACCGTTGGGAGACTCATT |  |  |
| GmActQF | CCCTCAACCCTAAAGCCAACA |  | Real-time PCR |
| GmActQR | CGGAGTCCAGCACGATACCA |  |  |
| GalQF | GAAGATCGCTTTCATAGTCGCA |  | Real-time PCR |
| GalQR | GTGGCTTGACGGTGATGGTTAC |  |  |
| RPL32QF | AAGCACTTCATCCGCCACC |  | Real-time PCR |
| RPL32QR | GCGACGCACTCTGTTGT |  |  |
| DrsQF | TACTTGTTCGCCCTCTTCGC |  | Real-time PCR |
| DrsQR | CATCCTTCGCACCAGCACTT |  |  |

**Table S2.** Comparative analysis of different fruit-fly lines infected by different fungal strains.

|  | **LT_50_ (days)** | **Log-rank test*** |
| --- | --- | --- |
| **WT infection** | | |
| W1118 | 3.5±0.04 | − |
| *PPO1^∆^* | 2.5±0.08 | χ^2^=52.21; *P=*4.99e-13 |
| *PPO2^∆^* | 3.5±0.05 | χ^2^=0.24; *P*= 0.62 |
| *PPO1^∆^PPO2^∆^* | 2.5±0.06 | χ^2^=67.21; *P*=2.44e-16 |
| **∆*MrM35-4* infection** | | |
| W1118 | 4.0±0.11 | − |
| *PPO1^∆^* | 3.0±0.06 | χ^2^=66.25; *P*=3.98e-16 |
| *PPO2^∆^* | 3.5±0.04 | χ^2^=13.64; *P*=2.21e-04 |
| *PPO1^∆^PPO2^∆^* | 2.5±0.05 | χ^2^=104.85; *P*=1.32e-24 |
| **WT::OE infection** | | |
| W1118 | 3.0±0.07 | − |
| *PPO1^∆^* | 3.0±0.07 | χ^2^=0.45; *P=*0.50 |
| *PPO2^∆^* | 3.5±0.04 | χ^2^=17.62; *P=*2.69e-05 |
| *PPO1^∆^PPO2^∆^* | 2.5±0.06 | χ^2^=45.92; *P=*1.23e-11 |

*, The comparison is made between wild-type and mutant flies for the infection with each fungal strain.


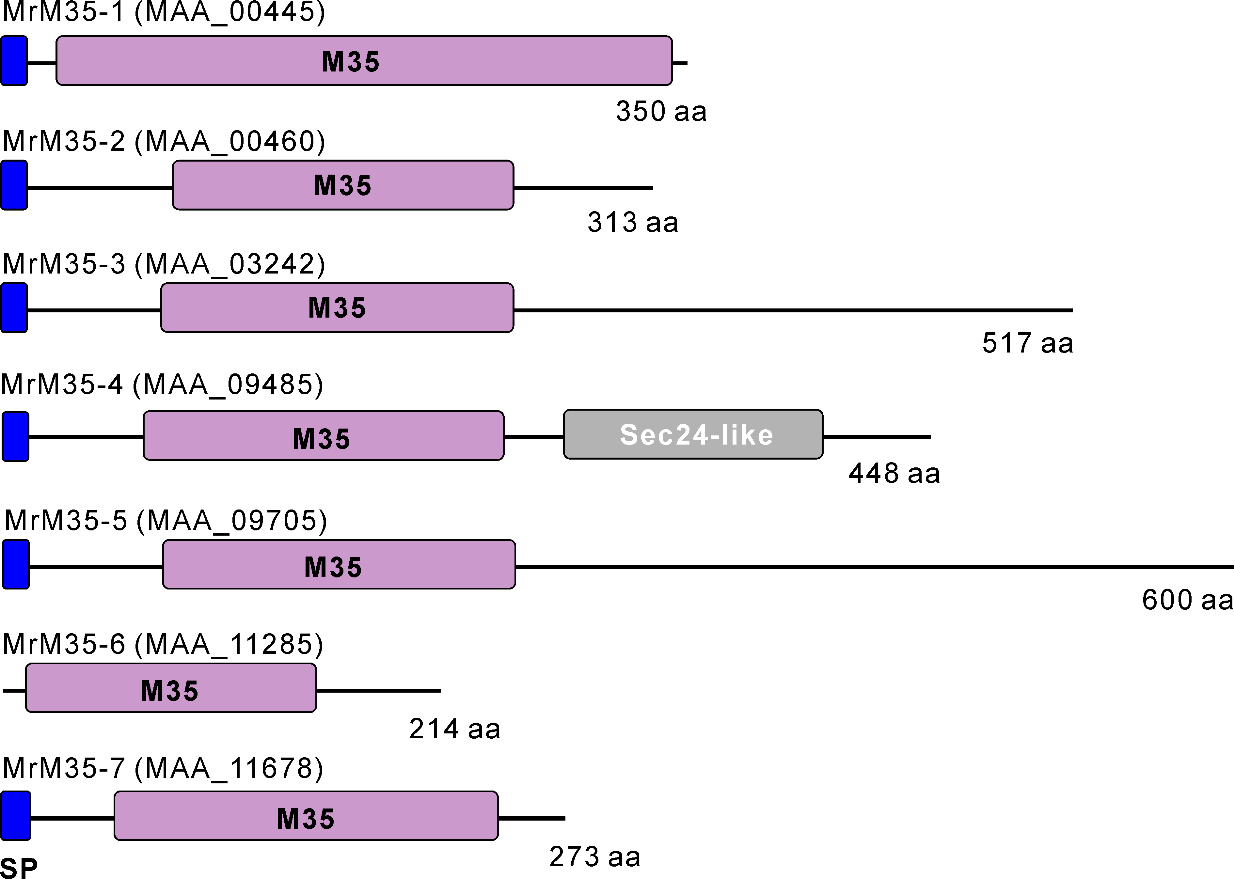


**Figure S1.** Schematic structuring of the seven M35 metalloproteases encoded in *Metarhizium robertsii*. SP, signal peptide.


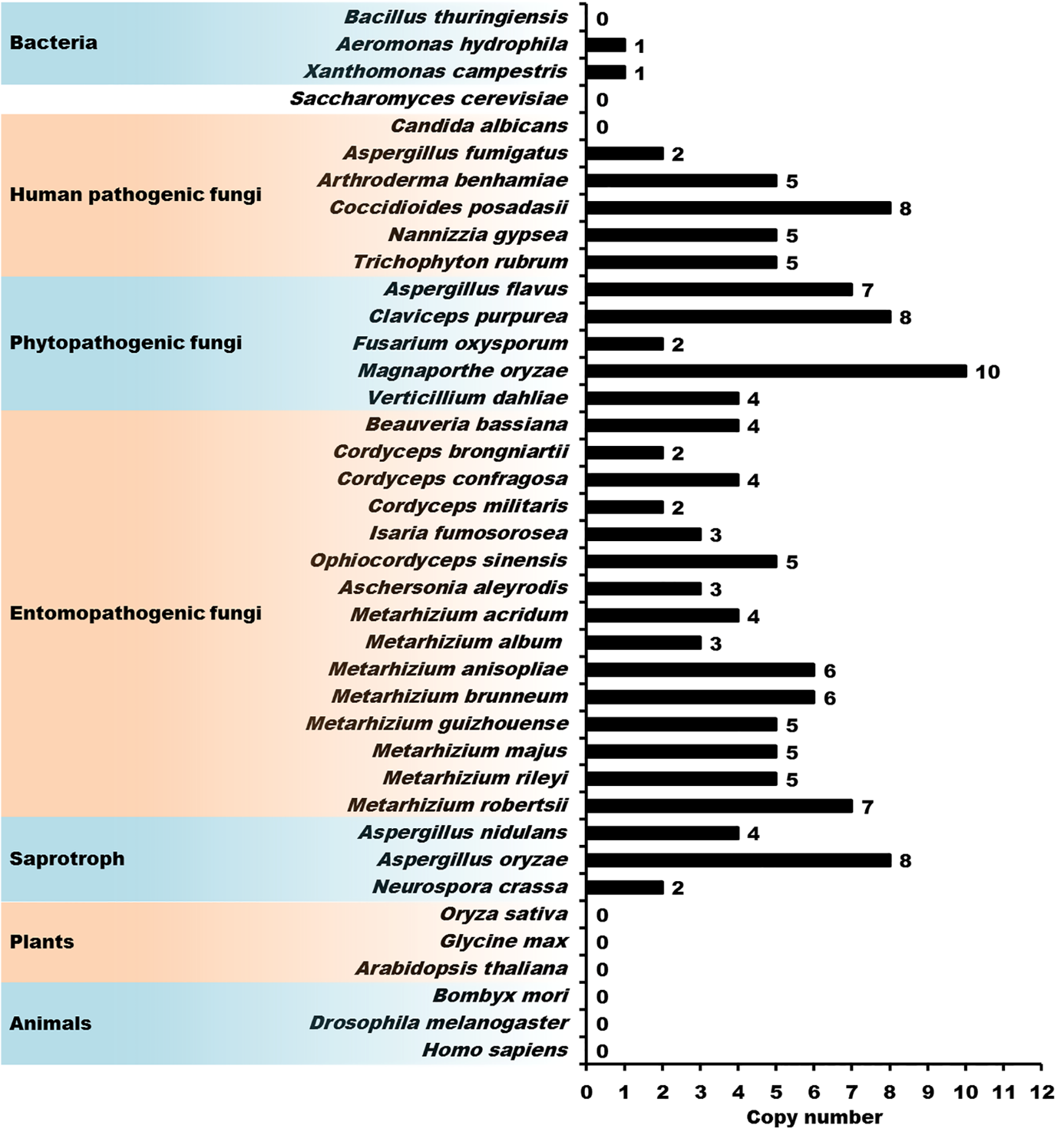


**Figure S2.** Number of M35 domain-containing proteins encoded in the genomes of selected organisms.





**Figure S3.** Phylogenetic analysis of the putative metalloproteases from the selected fungi and bacteria. The neighbor-joining tree was generated using the full amino acid sequence of each protein. The tree was generated using a Dayhoff model with a pairwise deletion of missing data/gaps and 500 replicates of bootstrapping. The MPs MrM35-1~MrM35-7 used in this study and those have been functionally studied are highlighted in bold. SF, subfamily.





**Figure S4.** Phylogenetic analysis of the M35 domains retrieved from the selected fungi and bacteria. The neighbor-joining tree was generated using the extracted M35 domain sequence from each protein. The tree was generated using a Dayhoff model with a pairwise deletion of missing data/gaps and 500 replicates of bootstrapping. The MPs MrM35-1~MrM35-7 used in this study and those have been functionally studied are highlighted in bold. dSF, domain-specific subfamily.





**Figure S5.** Verification of gene deletion, complementation and overexpression. (A) PCR verification of the deletion of seven MP genes and the complementation of ∆*MrM35-4*. The genomic DNA samples extracted from the wild-type (WT) and the randomly selected transformants were used as templates for PCR. (B) qRT-PCR validation of the randomly selected mutants to overexpress *MrM35-4*. The RNA samples extracted from the WT and randomly selected transformants harvested from a liquid medium were converted to cDNAs and used for qRT-PCR analysis. OE3 mutant was selected and used for further analysis.


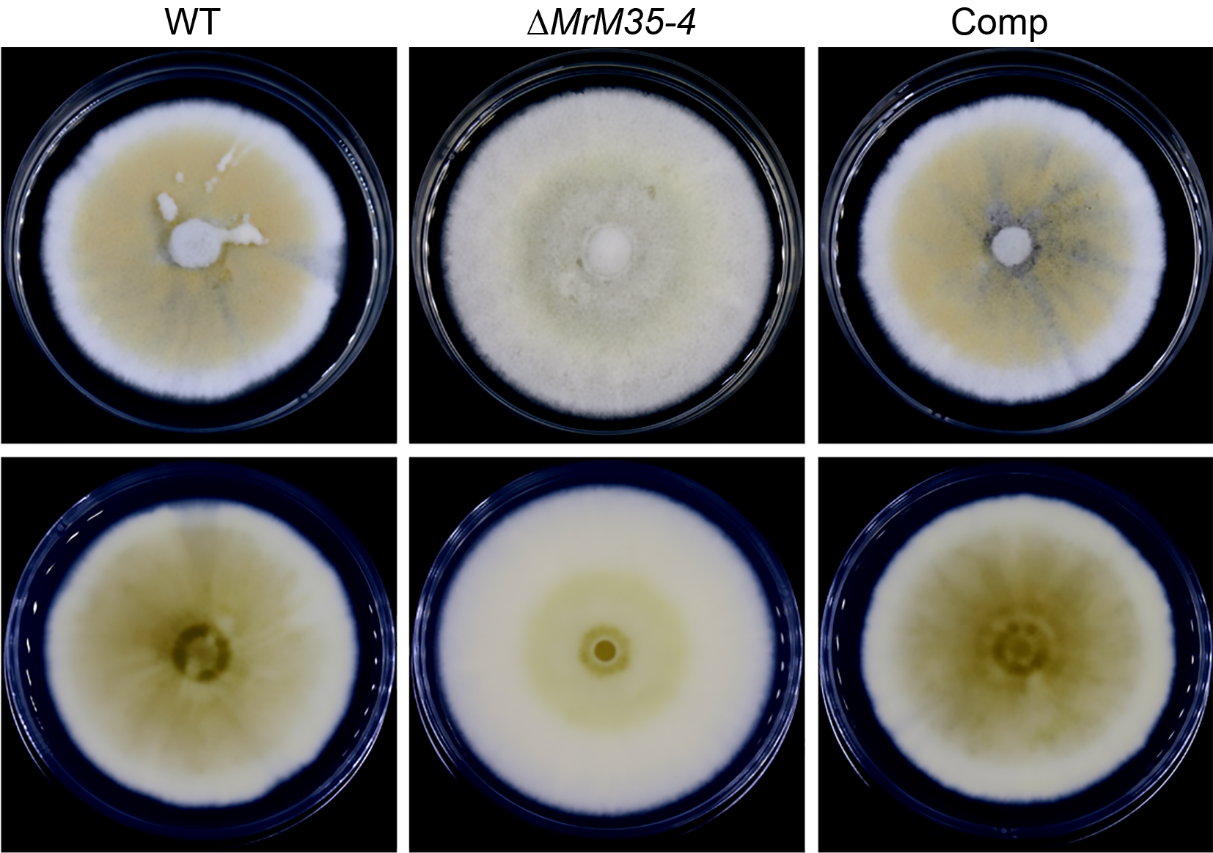


**Figure S6.** Culture phenotyping. Both the wild-type (WT) and mutants were grown on PDA for two weeks and then photographed. The lower panel shows the corresponding back features of each strain. Comp, the complemented mutant.


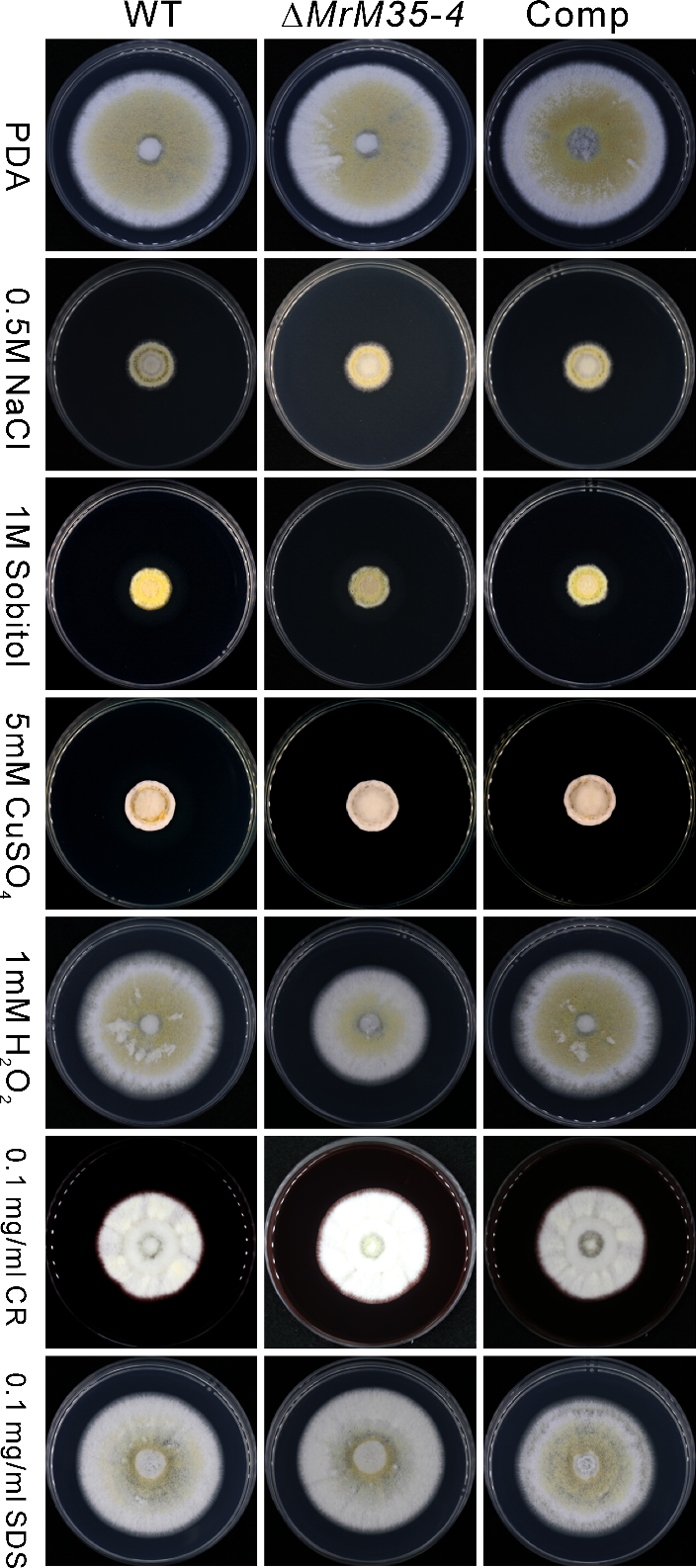


**Figure S7.** Stress response assays. Both the wild-type (WT) and mutants were grown on PDA or PDA amended with different compounds (shown in the right) for two weeks. Comp, the complemented mutant; CR, Congo red; SDS, sodium dodecyl sulfate.


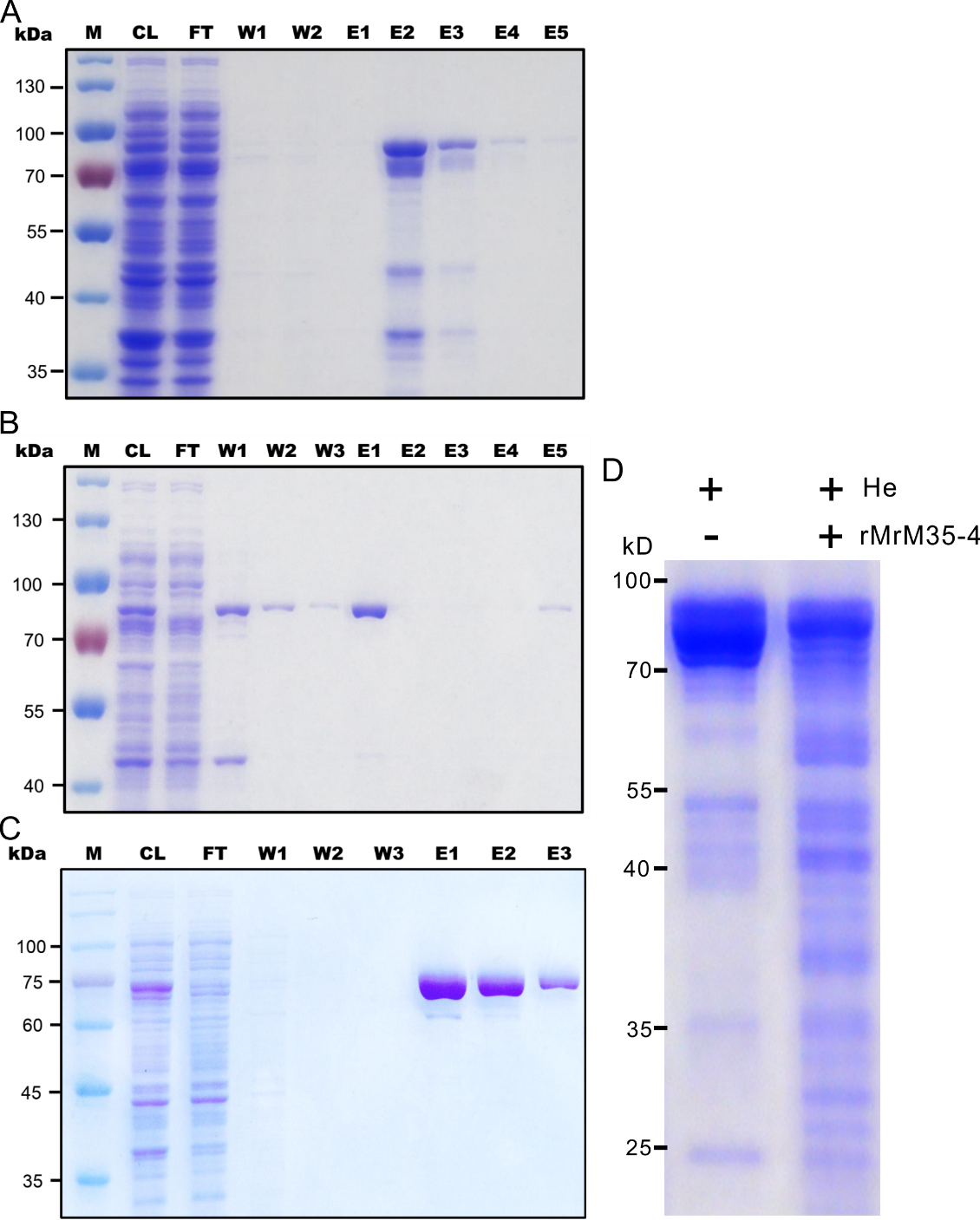


**Figure S8.** SDS-PAGE analysis of protein expression, purification and hemolymph digestion. (A) Expression and purification of rMrM35-4. (B) Expression and purification of rPPO1. (C) Expression and purification of rPPO1. M, protein marker; CL, cell lysate; FT, flow through; W1-3, sequential buffer washes; E1-5 or E1-3, different elutions. (D) Treatment of cell-free hemolymph with rMrM35-4. He, cell-free hemolymph of *G. mellonella*. In a 50 μl reaction system, 100 μg hemolymph protein was treated with or without 1 μg rMrM35-4 for one hour.
